# Tissue-Resident Memory T Cells in Antifungal Immunity

**DOI:** 10.3389/fimmu.2021.693055

**Published:** 2021-05-25

**Authors:** Salomé LeibundGut-Landmann

**Affiliations:** ^1^ Section of Immunology, Vetsuisse Faculty, University of Zürich, Zürich, Switzerland; ^2^ Institute of Experimental Immunology, University of Zürich, Zürich, Switzerland

**Keywords:** tissue-resident memory T cells (TRM), antifungal immunity, fungal commensals, pathogenic fungi, immunopathology, vaccine immunity (Min.5-Max. 8)

## Abstract

Fungi are an integral part of the mammalian microbiota colonizing most if not all mucosal surfaces and the skin. Maintaining stable colonization on these surfaces is critical for preventing fungal dysbiosis and infection, which in some cases can lead to life threatening consequences. The epithelial barriers are protected by T cells and additional controlling immune mechanisms. Noncirculating memory T cells that reside stably in barrier tissues play an important role for host protection from commensals and recurrent pathogens due to their fast response and local activity, which provides them a strategic advantage. So far, only a few specific examples of tissue resident memory T cells (TRMs) that act against fungi have been reported. This review provides an overview of the characteristics and functional attributes of TRMs that have been established based on human and mouse studies with various microbes. It highlights what is currently known about fungi specific TRMs mediating immunosurveillance, how they have been targeted in preclinical vaccination approaches and how they can promote immunopathology, if not controlled. A better appreciation of the host protective and damaging roles of TRMs might accelerate the development of novel tissue specific preventive strategies against fungal infections and fungi-driven immunopathologies.

## Introduction

Epithelial barriers cover the entire external and internal surfaces of our body to shield it from potentially harmful environmental influences, including toxic substances, radiation, and pathogens. Many of these barriers also fulfil essential physiological functions, such as nutrient uptake in the intestine or gas exchange in the lung. This entails challenging and partly opposing requirements for compartmentalization and permeability. One of the biggest challenges for barrier tissues is the maintenance of homeostasis with commensal microbes, while at the same time they have to prevent invasion and overgrowth of pathogenic microbes and the emergence of inflammatory disorders. Commensal microbes actively contribute to these processes, whereby the role of commensal fungi is increasingly being recognized (1–8). Stable maintenance of the microbiota in equilibrium with the host is strictly dependent on the immune system. Skin and mucosae harbor large numbers of myeloid and lymphoid immune cells that actively respond to commensal microbes, including commensal fungi (9–11), with T cells playing a predominant role in barrier tissue immunity against fungi. Thus, containment of the microbiota relies on active immunosurveillance and is not due to passive ignorance. In turn, the constant immune activation by the microbiota has to be tightly regulated to limit overt responses to innocuous microbes and to avoid tissue damaging inflammation. At the same time, the immune system must remain responsive against invading and disseminating pathogens.

The interest in barrier tissue immunity has steadily increased over the past decades, even though technical challenges have initially limited rapid advances in the field. Mucosal and cutaneous immune cells are scattered throughout the tissue parenchyma. They are often tightly associated with neighboring (non-immune) cells and their survival and function depends on the tissue environment, all of which hampers the isolation and ex vivo analysis of these cells (12). Access to human samples other than blood represents another limitation when studying human immunity in barrier tissues. Refined protocols for single cell isolation in combination with high-dimensional flow or mass cytometry have helped to define cellular subsets (13, 14). Single cell omics approaches, which can provide multidimensional high-resolution data (15–17), and advanced *in situ* imaging techniques, which provide spatial information for multiparameter settings (18) have further helped to promote the field. Moreover, refined genetic models allow selective targeting of specific cellular subsets in mice in a tissue- and/or time-restricted manner (19, 20). A novel humanized mouse model was recently reported to functionally study human cells *in situ* in the skin (21).

**Figure 1:**
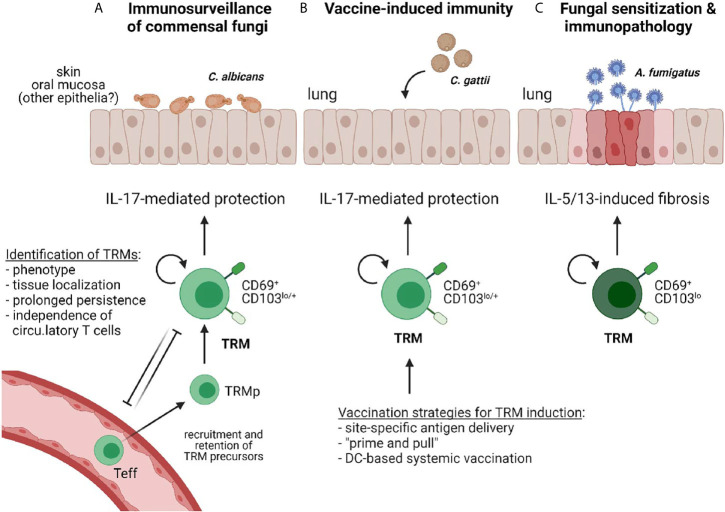
Reported examples of TRMs in antifungal immunity. **(A)** IL-17 producing CD4^+^ TRMs provide immunosurveillance against commensal fungi (e.g. *C. albicans*) that colonize barrier tissues such as the skin or the oral mucosa to prevent dysbiosis (23, 24). **(B)** Vaccine-induced immunity protects against fungal pathogens (e.g. *C. gattii*) *via* elicitation of IL-17-producing CD4^+^ TRMs (25). Different vaccination strategies for TRM induction in barrier tissues are indicated. **(C)** Antifungal TRMs can promote inflammation and immunopathology, such as those induced by *A. fumigatus* sensitization in the airways (26).

These approaches and combinations thereof have led to the discovery of various subsets of tissue-specific myeloid and lymphoid immune cells, including noncirculating memory T cells whose maintenance and function is regulated by tissue-specific environmental cues. These sessile memory T cells are referred to as tissue resident memory T cells (TRMs) (22). Seeding of the peripheral tissues with differentiated and long-lived T cells that are specialized in local microbe control is a major strategic advantage for both, the containment of commensal microbes and the rapid on-site control of (re)-appearing pathogens. To date, only a handful of papers have reported the existence of TRMs directed against fungi. In this review I discuss these studies in the context of the current general understanding of TRMs in antigen-specific protection from recurring pathogens and immunosurveillance of commensal microbes, but also of the pathological effects that they can exert. Moreover, I point out scenarios how TRMs might be harnessed for inducing vaccine immunity against fungi (**Figure 1**). Together, this review shall highlight the potential of this T cell subset in antifungal immunity in health and disease, and emphasize the gaps in knowledge that remain to be filled in the future.

## How Are TRMs Classified?

TRMs comprise CD4^+^ and CD8^+^ T cell subsets in mucosal tissues and the skin, but also in visceral organs, such as liver and lung. However, a T cell in any of these tissues is not automatically a TRM cell, even if it displays features of memory. TRM cells constitute a specific subset of memory T cells in non-lymphoid tissues, independent of circulating T cells. The following criteria define tissue-residency of T cells (27). First, TRMs can be identified by phenotyping: they express elevated levels of CD69, CD103, CD49a and CD11a when compared with circulating memory T cells, although none of these markers unequivocally denotes tissue residency and expression varies between CD4^+^ and CD8^+^ TRMs and between TRMs in different tissues. Second, the transcriptional signature can help distinguishing TRMs from other phenotypically similar cell subsets. Third, intravascular labelling allows identification of cells that are not in the circulation, at least at the time of analysis. Fourth, *in vivo* migration assays such as parabiotic surgery, *in situ* tissue labelling or tissue transplantation describe prolonged tissue persistence of T cells with limited recirculation potential. Finally, cutting the supply of circulatory T cells, for instance by blocking the migration of circulatory T cells or by selectively ablating them, disconnects the populations of TRM and circulatory T cells and thereby allows establishing autonomy of TRM survival and renewal. Each of these approaches has limitations, but together, they enabled the identification of TRMs as distinct non-recirculating long-lived sessile cells in various tissues (27).

In some tissues such as the epidermis of mice, CD8^+^ TRMs were found to display a slow crawling behavior and extend long protrusions in between their neighboring cells. This may help them to dynamically scan a large portion of their microenvironment for the presence of antigen (28, 29). Therefore, although tissue-residency follows strict criteria, it does not equal immobility within a given tissue. In contrast to CD8^+^ TRMs, their CD4^+^ counterparts were shown to form distinct microanatomical structures referred to as “memory lymphocyte clusters”, which are driven by TRM-derived cytokines (in particular IFN-γ) and chemokines derived from macrophages, both of which being central to cluster formation (30).

In compliance with all T cells TRMs possess a T cell receptor that determines their antigen-specificity. Until today, TRMs directed against diverse commensal and pathogenic microbes have been identified in the skin, the gastrointestinal tract, the reproductive tract, the nasal tissue, the lungs, and the liver. As such, fungi specific TRMs have also been reported: CD4^+^ TRMs responding to the commensal yeast *C. albicans* have been identified in healthy human skin based on phenotypic markers and cytokine production (23). In a murine model of *C. albicans* commensalism, the induction of fungus-specific Th17 cells with TRM features have been recapitulated (24). Beyond expression of CD69, CD103 and CD11a, *C. albicans*-specific TRMs also fulfil the criteria of TRMs to stay protected from *in vivo* labelling with an intravenously-injected CD4 antibody, and to be maintained independently of circulatory T cells (24). Another example of fungus-specific TRMs is provided by a study about *Aspergillus* sensitization in mice, where CD69^hi^ CD103^lo^ CD4^+^ TRMs were found induced in the lung, with separate subsets producing IL-5 and IL-17 (26). Their transcriptome profile and the finding that they were spared from the vasculature confirmed their identity. TRMs directed against other fungi await to be identified, especially those that respond to commensal and ubiquitous fungi, which are in constant contact with our barrier tissues and against which lasting T cell immunity is a prerequisite of homeostasis. The presence of circulatory memory CD4^+^ T cells reactive against diverse fungi in the healthy human blood (1) suggests that corresponding TRM subsets with the same specificities will likely be present in the epithelial tissues.

## What Are the Functional Attributes of TRMs?

The raison d’être of memory T cells is to direct rapid and efficient protection against previously encountered antigens, which applies to TRMs as well. The first report on the function of TRMs in 2011 described the relevance of influenza-specific lung-resident memory CD4^+^ T cells for *in situ* protection against respiratory viral challenge, as they mediate enhanced viral clearance and survival to lethal influenza infection when compared to recirculating memory CD4^+^ T cells (31). Over the past ten years, the protective capabilities of CD4^+^ and CD8^+^ TRM cells have been demonstrated for members of all pathogen classes, including fungi, predominantly in murine infection models (23, 31–43). In humans, the protective effect of TRMs is evidenced by the correlation between presence of pathogen specific TRMs and enhanced protectivity (44–46).

The results from human studies emphasize that TRMs not only provide rapid infection control after re-exposure to a previously encountered and combatted pathogen, a setting modelled by many experimental mouse infection studies (N.B.: in SPF mice, most experimentally administered infectious agents are rapidly cleared), but that TRMs are also particularly critical for host protection during persistent infection (22). This in turn suggests that TRMs likely contribute to immunosurveillance of commensal microbes to which the host is constantly exposed. Indeed, TRMs with reactivity to intestinal microbes are abundant in the gut tissue of healthy individuals (47). An involvement of TRMs in maintaining homeostasis and preventing overgrowth of commensal fungi is evidenced by *C. albicans*-responsive CD4^+^ TRMs identified in healthy human skin (23) and in an experimental model of persistent *C. albicans* colonization in the oral mucosa of mice (24). In both species, these cells produce IL-17 in response to fungal re-stimulation, in line with the well-recognized host protective role of IL-17 against *C. albicans* in barrier tissues (48).

Overall, the population of TRMs in a given tissue reflects its history of exposures to pathogenic and commensal microbes. Analysis of T cell subsets in the intestinal mucosa of children revealed that naïve recent thymic emigrants and effector memory T cells predominate during early life, whereas TRMs progressively accumulate with increasing age (49). The continuous build-up thereby results in specific TRM pools that are geared to the need of the specific sites. By orchestrating regional immune responses to ongoing microbial exposure in a clinically silent manner, TRMs act as central players in maintaining tissue homeostasis and a stable host-microbe equilibrium.

## How Do TRMs Exert Their Functions?

Like all T cells, TRMs respond to TCR-mediated stimulation and activate effector functions in an antigen-specific manner. The advantage of TRMs, if compared to circulatory T cell subsets, arises from their strategic positioning within tissues, which allows immediate local recognition of infected or antigen presenting cells and a rapid recall of canonical effector functions, such as cytokine secretion or perforin- and granzyme-mediated target cell killing (22). Thereby, TRMs can prevent invasion and dissemination of microbes that have crossed the physical barrier through which they are usually segregated, and thwart clinically apparent infection.

Upon re-encounter with cognate antigen, TRMs can profoundly alter the local tissue environment. Through production of cytokines, they can trigger rapid adaptive and innate immune responses, including local humoral responses, activation of local innate immune cells (dendritic cells, NK cells) and recruitment of circulating lymphocytes, resulting in a remarkable cross-over between innate and adaptive immunity. Thereby, the triggering of a small number of TRMs is amplified into an organ-wide response, which can provide protection against even antigenically unrelated organisms (50, 51). This may offer a particular advantage for controlling escape variants of pathogens that might emerge.

An explanation for the reactivity against unrelated antigens is provided by the broad cross-reactivity of the T cell repertoire (52). Widely cross-reactive T cells are abundant in the human T cell memory compartment comprising T cells that react against antigens to which the host has not been exposed previously (52, 53). Cross-reactivity has also been proposed for *C. albicans*-responsive memory T cells to explain the diverse and systemic effects that these cells can exert (54). In animal models, TRMs have further been found to respond to noncognate, bystander activation (55–57). The potential of microbiota-specific cross-reactive TRMs for host protection is illustrated by skin-resident TRMs induced in response to topically applied *Staphylococcus epidermidis*, which specifically reinforce the barrier function of the epithelium and prevents overgrowth of heterologous microbes, including *C. albicans*, in a CD8^+^ T cell- and IL-17-dependent manner (58). In addition to TCR cross-reactivity, TRMs can also provide heterologous protection by responding to bystander activation *via* TCR-independent mechanisms (59).

Besides their potential broad reactivity, TRMs may display some degree of functional plasticity. In fact, skin-resident commensal-specific memory Th17 cells were found to rapidly activate a type 2 effector program in response to tissue injury (60). This cell-intrinsic flexibility might allow them to swiftly adapt to environment insults, which might be caused by physical damage or invasive pathogens.

## How Are TRMs Implicated in Immunopathological Responses?

As a consequence of their ability to rapidly respond to antigen or to react even in an antigen-unspecific manner, and to display functional plasticity, TRMs may entail severe consequences on tissue homeostasis. Indeed, commensal-specific responses in barrier tissues are characterized by the production of IL-17, a cytokine that promotes antimicrobial functions and barrier integrity of epithelia (48). IL-17 production in barrier tissues is particularly relevant for immunosurveillance of *Candida*, as genetic defects in genes of the IL-17 pathway drive the development of chronic mucocutaneous candidiasis (10). However, IL-17 can also contribute to the etiology and pathology of various inflammatory skin disorders, such as psoriasis (61).

Psoriasis is a T cell mediated disease (62). Although the specific antigens targeted by psoriatic T cells remain undefined (63), skin-colonizing microbes are likely candidates. Strikingly, psoriatic lesions are limited to characteristic sites, such as the flexor sides of extremities, the sacral region, and soles of the feet, and disease recurs at always the same predilection sites. An explanation for the site-specific manifestation of disease may be provided by tissue residency of the disease-mediating T cells. Indeed, a study looking into lesional and non-lesional skin samples from psoriasis patients found that the epidermis with active psoriasis was massively infiltrated by CD8^+^ TRMs when compared to non-lesional skin and healthy skin, with a 100-fold increase of TRMs in active psoriatic lesions (64, 65). The CD103^+^CD8^+^ T cells in psoriatic skin expressed IL-17 and IL-22 mRNA. Importantly, IL17A-expressing CD103^+^CD8^+^ T cells were found to be present in both active psoriatic plaques as well as in resolved skin lesions, but not in healthy control skin (65, 66), suggesting that they are poised for re-initiation of inflammation. Therefore, TRMs may act as drivers of disease flares in a site-specific manner.

TRMs were also implicated in other chronic and recurring inflammatory disorders that are characterized by localized manifestations in the skin, the oral-gastrointestinal tract, or the respiratory tract. For instance, in patients with chronic rhinosinusitis, TRMs accumulate in the nasal polyps (67). In periodontitis, expansion of Th17 TRMs drives immunopathology in response to local dysbiosis (68). In vitiligo, a pigmentation defect of the skin mediated by melanocyte eradicating cytotoxic T cells (69), the proportion of perforin and granzyme B expressing CD8^+^CD103^+^ TRMs is increased in lesional compared to non-lesional and healthy skin (70). Similarly, in atopic dermatitis (AD), CD69^+^CD103^+^ TRMs were significantly expanded in lesional compared to non-lesional skin and healthy controls (71). In AD, the chronic allergic skin inflammation is dominated by Th2 cells that react against environmental allergens such as house dust mite proteins, but also skin microbes such as *S. aureus* and the skin commensal yeast *Malassezia* (72). AD lesions occur primarily on the cubital and popliteal fossae, and in head and neck type AD on the upper trunk, shoulders, and scalp, with age-related variations (72). Certain subtypes of AD display overlapping characteristics with psoriasis, especially by the involvement of IL-17-producing T cells (73). However, AD and psoriasis are clearly separable diseases, not least based on the different skin regions that are affected (73, 74). A likely explanation for the site-specific recurrence of the lesions may once again be provided by the limited distribution of pathogenic TRMs across different skin sites, and by their diverging antigen specificity (63).

TRMs have also been linked to the pathogenesis of inflammatory bowel disease (IBD). CD4^+^CD69^+^ TRMs producing pro-inflammatory cytokines were found enriched in the intestinal mucosa of IBD compared to control patients, and the presence of these cells was predictive of the development of flares (19). Attenuation of disease in an experimental model of transfer colitis when Hobit- and Blimp-1-deficient CD4^+^ T cells were transferred, confirmed the disease-driving role of CD4^+^ TRMs in IBD (19). Finally, TRMs can also mediate immunopathology in the airways (75). In experimental allergy models, lung CD4^+^ TRMs generated in response to allergen exposure can promote reactive airway disease (76, 77). Similarly, in a model of fungal allergy, sensitization with *Aspergillus fumigatus* induced a population of CD103^lo^CD69^hi^ CD4^+^ TRMs that promoted pathology through enhanced production of IL-5 and IL-13 in the lung. Of interest, the fibrotic response was ameliorated by CD103^hi^ tissue resident regulatory T cells (26).

The detrimental consequences that TRMs can have in chronic inflammatory diseases emphasize the need for tight regulation of their activity. Recent experimental evidence suggests that TRM intrinsic mechanisms control their reactivation to prevent damaging immunopathology. In a model of contact hypersensitivity to 2,4-dinitrofluorobenzene, inhibitory checkpoint receptor antagonists dramatically enhanced the magnitude and severity of eczema exacerbations (78). Similarly, the antiviral activity of TRMs against HSV-1 re-activation in latently infected rabbits was restored by antibodies directed against PD-1 and LAG-3 (79). This indicates that TRM responsiveness is restrained during steady state. In support of this hypothesis, TRMs express various inhibitory receptors including PD-1, CLTA-4, LAG-3 amongst others (80, 81). Moreover, tissue residency of T cells was associated with an immunoregulatory gene expression program, including IL-10 cytokine expression under the control of the transcriptional regulator c-MAF (82). Such cell-intrinsic regulatory mechanisms likely exist also for TRMs directed against fungi to ensure that under homeostatic conditions they remain clinically silent even if they are constantly exposed to stimulation by commensal and environmental fungi, although specific examples are not available yet.

## How Can TRMs Be Harnessed for Achieving Vaccine Immunity?

The important role of TRMs in site-specific protection renders them an attractive target in vaccine development, particularly against tissue-tropic infections. This also applies to vaccines against fungal agents. While induction of long-lived immune memory against a vaccine antigen represents a challenge by itself, induction of residency adds another dimension of complexity to vaccine design. Site specific antigen delivery has produced promising results in preclinical studies with regards to the induction of TRM and vaccine immunity. As such, nasal immunization with certain antigen/adjuvant combinations results in effective induction of TRMs in the respiratory tract (83–85). Another example is provided by the intradermal injection of a synthetic DNA vaccine encoding a *Leishmania* antigen, which resulted in enhanced protection from experimental infection with *Leishmania major* in the skin if compared to intramuscular vaccination (86). Vaccine-induced TRMs have also proven effective in immunotherapy against melanoma in an experimental tumor model (87). Despite these promising proof of principle studies, TRM induction in barrier tissues by site-specific vaccine delivery remains a challenge due to the high tolerance threshold in these organs (88). Transient microbiota depletion has recently been explored for temporarily restraining colonization resistance in the gut and thereby allowing expansion of a pool of antigen specific functional TRMs directed against orally delivered *Listeria monocytogenes*, which ultimately enhance protection against infectious re-challenge (89).

An alternative vaccination strategy for generating TRM-mediated vaccine immunity at relevant tissue sites has been explored by a two-step approach, which consists of first generating a systemic immune response against a vaccine antigen, for instance by intramuscular immunization, and then applying a localized inflammatory stimulus to ‘pull’ the newly primed T cells towards a specific tissue. This “prime and pull” strategy relies on the notion that TRM precursors are primed in secondary lymphoid organs before they migrate to peripheral tissue, where micro-environmental cues then drive TRM maturation (90–93). In case of CD8^+^ TRMs, adoption of residency has been described to depend on local TGF-β and IL-15 (94–97). TGFβ appears to be required at multiple steps in TRM development (98). In the skin, exposure of CD8^+^ T cells to TGF-β efficiently induces expression of CD103, which anchors TRMs to E-cadherin expressing epithelial cells (43, 81, 95, 96). Accumulation and persistence of TRMs in the epithelial niche is further determined by the downregulation of pathways linked to T cell egress from peripheral tissues and the upregulation of tissue-retention and pro-survival molecules (22), which are under the control of the transcription factors Hobit and Runx3 (99, 100), albeit with species-specific variations (19, 99). Long-term persistence of TRMs is thought to depend on IL-15 (94) and was in addition recently found to depend on continuous availability of autocrine TGF-β (101).

Although inflammatory stimuli such as imiquimod have been shown sufficient to “pull” TRM precursors to peripheral tissues, conversion of recruited T cells into TRMs may be maximized by local antigen recognition, especially in the lung (102, 103). If more generally valid, this notion might be highly relevant for fungal vaccination strategies, considering that memory T cells directed against many different fungi exist in healthy human blood (1). “Pulling” these pre-existing fungus-specific memory T cells to a specific tissue site for local protection from fungal overgrowth may provide a suitable approach under certain conditions.

Induction of TRM immunity against antigens to which the host was not previously exposed and to which the host has therefore not yet mounted a T cell response, was also achieved by alternative approaches, for instance by antibody targeting. Selective delivery of antigen to respiratory dendritic cells resulted in the development of lung CD8^+^ TRMs that were highly protective against lethal influenza challenge (104). Similarly, CD4^+^ TRMs were efficiently induced by a dendritic cell-based vaccine in the context of a pulmonary mycosis. Systemically administered antigen-loaded dendritic cells were found to migrate with high efficiency to the lung, where they elicited a local and resident CD4^+^ T cell response. The vaccine suppressed fungal burden in the lungs and improved the survival of mice infected with the highly virulent fungus *Cryptococcus gattii* (25).

Although site-specific accumulation of TRMs has been associated with successful vaccine immunity, induction of TRMs does not always equal resistance to infection, as demonstrated by the following study, which explored a fungal vaccination strategy in a preclinical model of *Blastomyces* infection. Intranasal administration of a subunit vaccine composed of *Blastomyces* endoglucanase-2 (Bl-Eng2), which harbors both, a Dectin-2 agonistic ligand and a CD4^+^ T cell epitope, induced a large number of tetramer^+^ TRMs in the lung of mice. However, it failed to protect against lethal infection with *Blastomyces* (105). In contrast, systemic administration of the same vaccine induced a population of tetramer^+^ CD4^+^ migratory T cells enriched within the pulmonary vasculature that migrated to the lung tissue upon challenge and efficiently protected mice against infection (105). While the underlying cause of the mucosal vaccine failure remains to be established, one hypothesis concerns the localization of the pathogen to be combatted by intranasally induced vaccine immunity, which is intracellular in case of many of the examples provided above, but extracellular in case of *Blastomyces*. A lot remains to be learned about the signals favoring induction of functional TRMs capable of conveying enhanced local protection and how this knowledge can be translated to make it applicable in vaccine design.

## Concluding Remarks

Over the past years the interest in TRMs has steadily grown and numerous studies have contributed to our current appreciation of the relevance of these cells residing in most, if not all non-lymphoid tissues where they contribute to tissue homeostasis and microbial control but can also drive immunopathology. In the field of fungal immunity, only very few reports are currently available on TRMs with antifungal functions. Fungus-specific TRMs follow the principles that were delineated for TRMs directed against other microbes and viruses. Fungi-specific TRMs are expected to populate all tissues that are naturally exposed to commensal or environmental fungi. Their exact features and their relevance in fungal control awaits to be identified, including similarities and differences between TRMs directed against distinct fungi and in different tissues. Besides promoting immunosurveillance, fungi specific TRMs will likely also be found to be involved in inflammatory pathologies of barrier tissues. More research on TRMs in fungal immunity using preclinical models and human tissues is warranted. The expected knowledge gain will help taking informed decisions for harnessing TRMs in antifungal vaccine development.

## Author Contributions

The author confirms being the sole contributor of this work and has approved it for publication.

## Funding

Work in the SL-L-lab is supported by the Swiss National Science Foundation (grant 310030_166206, 310030_189255 and CRSII5_173863).

## Conflict of Interest

The author declares that the research was conducted in the absence of any commercial or financial relationships that could be construed as a potential conflict of interest.
